# Supplementary material for: Analysis of the Association between Female Medical History and Thyroid Cancer in Women: A Cross-Sectional Study Using KoGES HEXA Data
Source: Int J Environ Res Public Health. 2021 Jul 29;18(15):8046. doi: 10.3390/ijerph18158046 (PMC8345436; doi:10.3390/ijerph18158046)
Supplement: Supplementary file 1 [file ijerph-18-08046-s001.zip › ijerph-1284945-supplementary.pdf]

Supplement Table S1 Analyses of the association between hysterectomy and oophorectomy

	Hysterectomy		P-value
	No	Yes	
Oophorectomy (n, %)			<0.001*
No	95,263 (99.2)	10,146 (89.8)	
Unilateral	718 (0.7)	697 (6.2)	
Bilateral	89 (0.1)	452 (4.0)	

*Chi-square test, Statistical significance at $P < 0.05$
